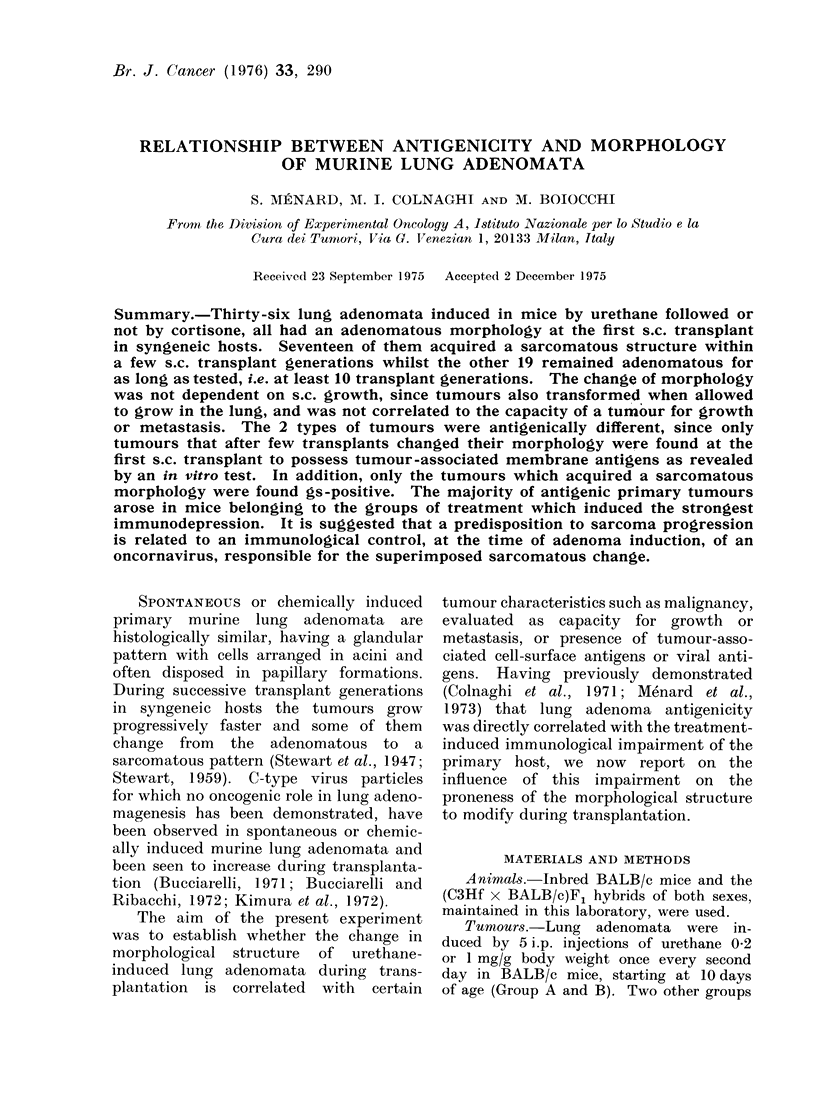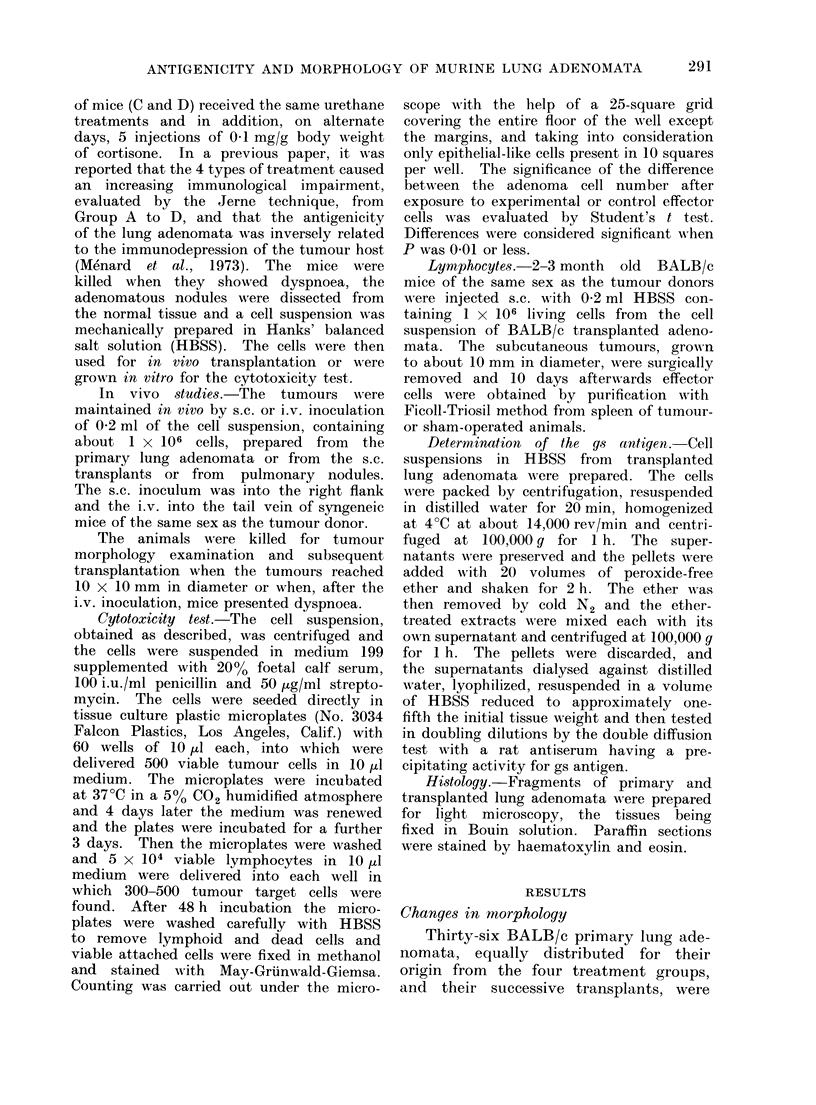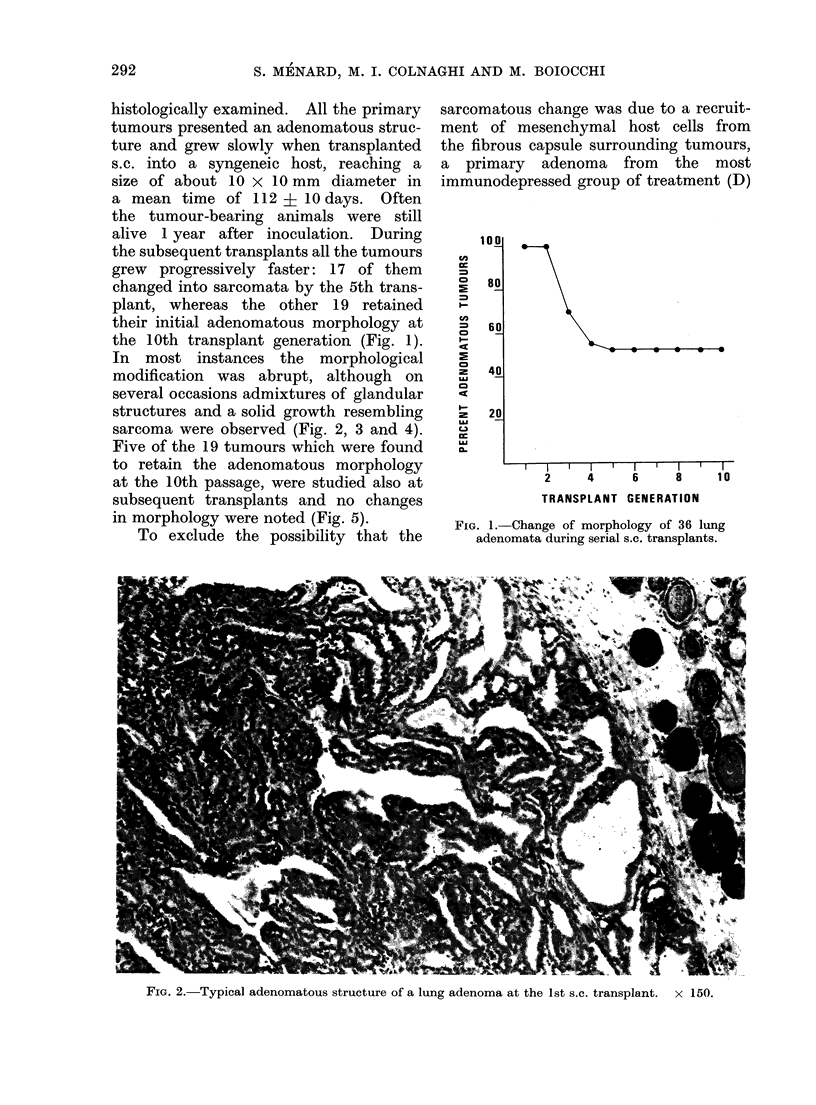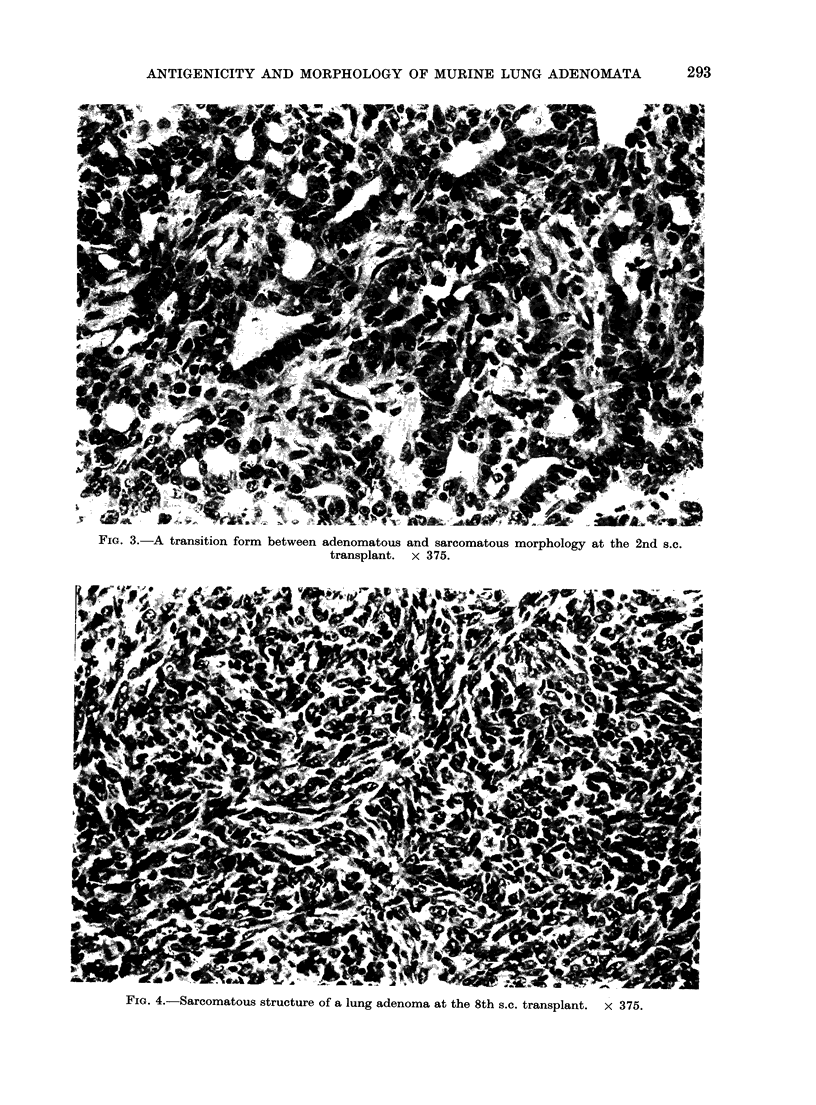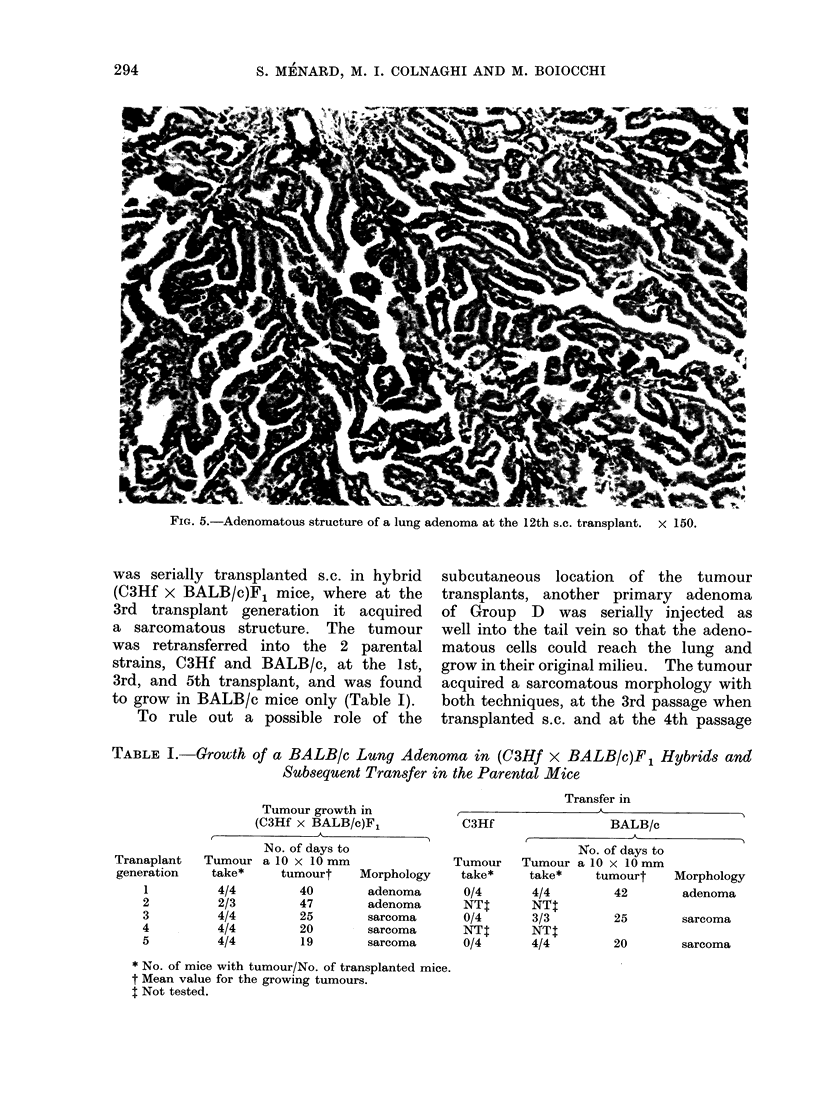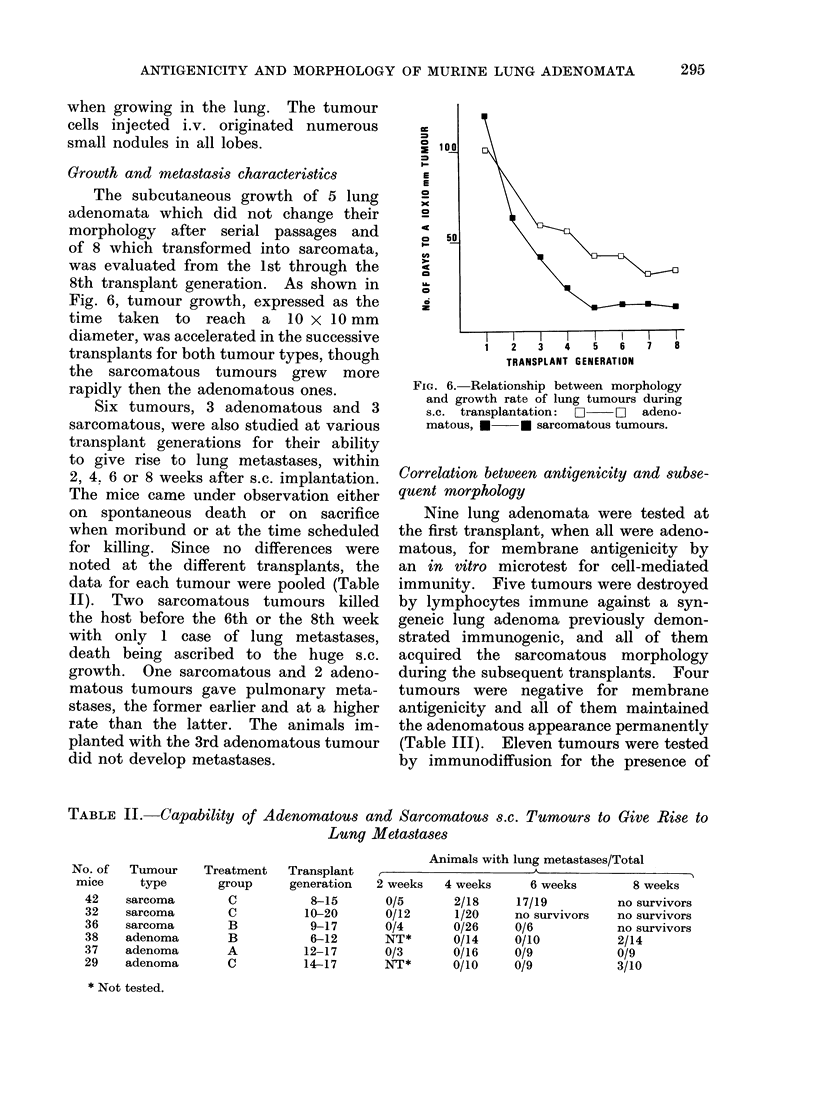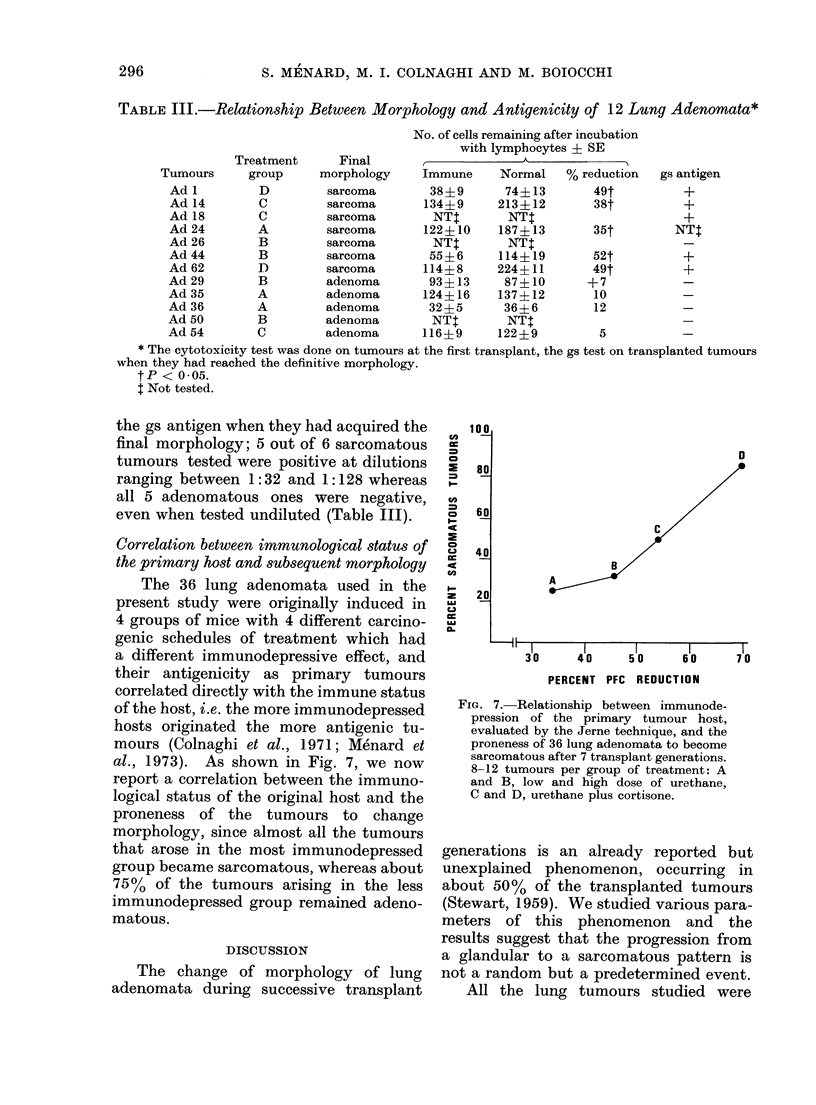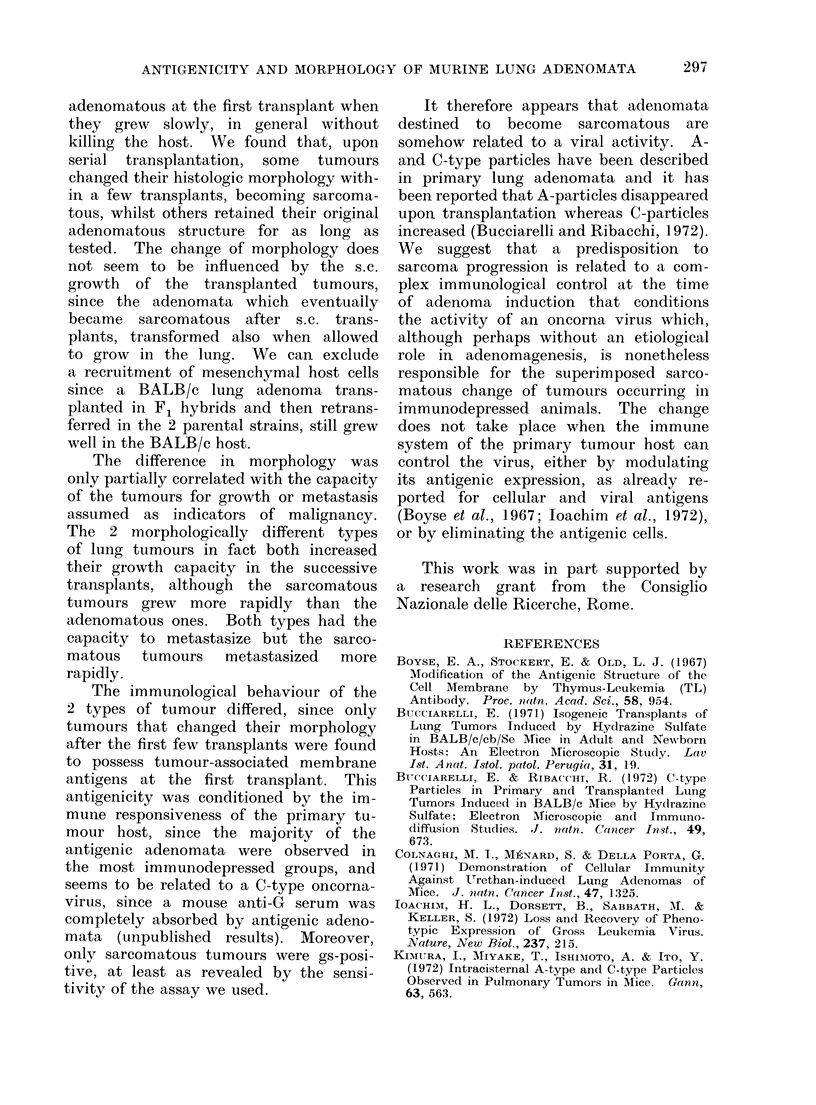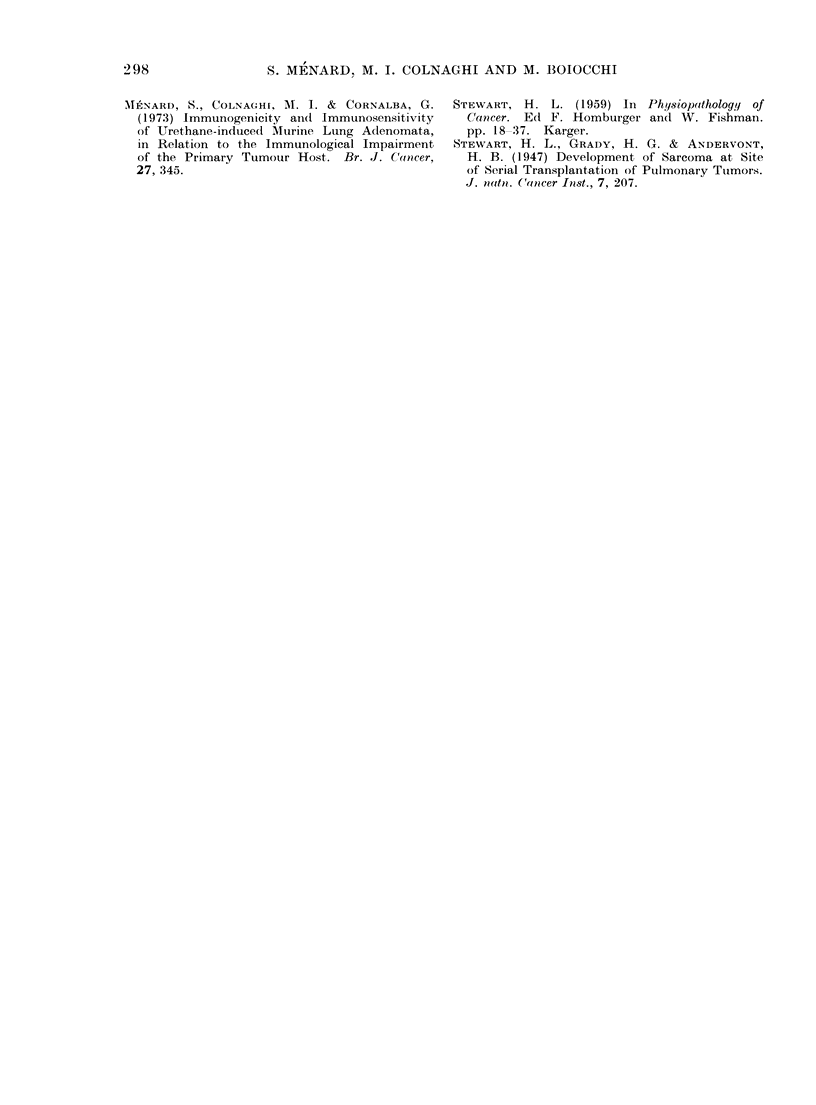# Relationship between antigenicity and morphology of murine lung adenomata.

**DOI:** 10.1038/bjc.1976.42

**Published:** 1976-03

**Authors:** S. Ménard, M. I. Colinaghi, M. Boiocchi

## Abstract

**Images:**


*Br. J. Cancer* (1976) **33**, 290

### RELATIONSHIP BETWEEN ANTIGENICITY AND MORPHOLOGY OF MURINE LUNG ADENOMATA

S. MÉNARD, M. I. COLNAGHI AND M. BOIOCCHI

*From the Division of Experimental Oncology A, Istituto Nazionale per lo Studio e la Cura dei Tumori, Via G. Venezian 1, 20133 Milan, Italy*

Received 23 September 1975 Accepted 2 December 1975

**Summary.—Thirty-six lung adenomata induced in mice by urethane followed or not by cortisone, all had an adenomatous morphology at the first s.c. transplant in syngeneic hosts. Seventeen of them acquired a sarcomatous structure within a few s.c. transplant generations whilst the other 19 remained adenomatous for as long as tested, *i.e.* at least 10 transplant generations. The change of morphology was not dependent on s.c. growth, since tumours also transformed when allowed to grow in the lung, and was not correlated to the capacity of a tumour for growth or metastasis. The 2 types of tumours were antigenically different, since only tumours that after few transplants changed their morphology were found at the first s.c. transplant to possess tumour-associated membrane antigens as revealed by an *in vitro* test. In addition, only the tumours which acquired a sarcomatous morphology were found gs-positive. The majority of antigenic primary tumours arose in mice belonging to the groups of treatment which induced the strongest immunodepression. It is suggested that a predisposition to sarcoma progression is related to an immunological control, at the time of adenoma induction, of an oncornavirus, responsible for the superimposed sarcomatous change.**

SPONTANEOUS or chemically induced primary murine lung adenomata are histologically similar, having a glandular pattern with cells arranged in acini and often disposed in papillary formations. During successive transplant generations in syngeneic hosts the tumours grow progressively faster and some of them change from the adenomatous to a sarcomatous pattern (Stewart *et al.*, 1947; Stewart, 1959). C-type virus particles for which no oncogenic role in lung adenomagenesis has been demonstrated, have been observed in spontaneous or chemically induced murine lung adenomata and been seen to increase during transplantation (Bucciarelli, 1971; Bucciarelli and Ribacchi, 1972; Kimura *et al.*, 1972).

The aim of the present experiment was to establish whether the change in morphological structure of urethane-induced lung adenomata during transplantation is correlated with certain tumour characteristics such as malignancy, evaluated as capacity for growth or metastasis, or presence of tumour-associated cell-surface antigens or viral antigens. Having previously demonstrated (Colnaghi *et al.*, 1971; Ménard *et al.*, 1973) that lung adenoma antigenicity was directly correlated with the treatment-induced immunological impairment of the primary host, we now report on the influence of this impairment on the proneness of the morphological structure to modify during transplantation.

### MATERIALS AND METHODS

*Animals.*—Inbred BALB/c mice and the (C3Hf × BALB/c)$F_1$ hybrids of both sexes, maintained in this laboratory, were used.

*Tumours.*—Lung adenomata were induced by 5 i.p. injections of urethane 0·2 or 1 mg/g body weight once every second day in BALB/c mice, starting at 10 days of age (Group A and B). Two other groups of mice (C and D) received the same urethane treatments and in addition, on alternate days, 5 injections of 0·1 mg/g body weight of cortisone. In a previous paper, it was reported that the 4 types of treatment caused an increasing immunological impairment, evaluated by the Jerne technique, from Group A to D, and that the antigenicity of the lung adenomata was inversely related to the immunodepression of the tumour host (Ménard *et al.*, 1973). The mice were killed when they showed dyspnoea, the adenomatous nodules were dissected from the normal tissue and a cell suspension was mechanically prepared in Hanks' balanced salt solution (HBSS). The cells were then used for *in vivo* transplantation or were grown *in vitro* for the cytotoxicity test.

In vivo *studies*.—The tumours were maintained *in vivo* by s.c. or i.v. inoculation of 0·2 ml of the cell suspension, containing about $1 \times 10^6$ cells, prepared from the primary lung adenomata or from the s.c. transplants or from pulmonary nodules. The s.c. inoculum was into the right flank and the i.v. into the tail vein of syngeneic mice of the same sex as the tumour donor.

The animals were killed for tumour morphology examination and subsequent transplantation when the tumours reached $10 \times 10$ mm in diameter or when, after the i.v. inoculation, mice presented dyspnoea.

*Cytotoxicity test*.—The cell suspension, obtained as described, was centrifuged and the cells were suspended in medium 199 supplemented with 20% foetal calf serum, 100 i.u./ml penicillin and 50 $\mu$g/ml streptomycin. The cells were seeded directly in tissue culture plastic microplates (No. 3034 Falcon Plastics, Los Angeles, Calif.) with 60 wells of 10 $\mu$l each, into which were delivered 500 viable tumour cells in 10 $\mu$l medium. The microplates were incubated at 37°C in a 5% $CO_2$ humidified atmosphere and 4 days later the medium was renewed and the plates were incubated for a further 3 days. Then the microplates were washed and $5 \times 10^4$ viable lymphocytes in 10 $\mu$l medium were delivered into each well in which 300–500 tumour target cells were found. After 48 h incubation the microplates were washed carefully with HBSS to remove lymphoid and dead cells and viable attached cells were fixed in methanol and stained with May-Grünwald-Giemsa. Counting was carried out under the microscope with the help of a 25-square grid covering the entire floor of the well except the margins, and taking into consideration only epithelial-like cells present in 10 squares per well. The significance of the difference between the adenoma cell number after exposure to experimental or control effector cells was evaluated by Student's *t* test. Differences were considered significant when *P* was 0·01 or less.

*Lymphocytes*.—2–3 month old BALB/c mice of the same sex as the tumour donors were injected s.c. with 0·2 ml HBSS containing $1 \times 10^6$ living cells from the cell suspension of BALB/c transplanted adenomata. The subcutaneous tumours, grown to about 10 mm in diameter, were surgically removed and 10 days afterwards effector cells were obtained by purification with Ficoll-Triosil method from spleen of tumour- or sham-operated animals.

*Determination of the gs antigen*.—Cell suspensions in HBSS from transplanted lung adenomata were prepared. The cells were packed by centrifugation, resuspended in distilled water for 20 min, homogenized at 4°C at about 14,000 rev/min and centrifuged at 100,000 *g* for 1 h. The supernatants were preserved and the pellets were added with 20 volumes of peroxide-free ether and shaken for 2 h. The ether was then removed by cold $N_2$ and the ether-treated extracts were mixed each with its own supernatant and centrifuged at 100,000 *g* for 1 h. The pellets were discarded, and the supernatants dialysed against distilled water, lyophilized, resuspended in a volume of HBSS reduced to approximately one-fifth the initial tissue weight and then tested in doubling dilutions by the double diffusion test with a rat antiserum having a precipitating activity for gs antigen.

*Histology*.—Fragments of primary and transplanted lung adenomata were prepared for light microscopy, the tissues being fixed in Bouin solution. Paraffin sections were stained by haematoxylin and eosin.

### RESULTS

### *Changes in morphology*

Thirty-six BALB/c primary lung adenomata, equally distributed for their origin from the four treatment groups, and their successive transplants, were histologically examined. All the primary tumours presented an adenomatous structure and grew slowly when transplanted s.c. into a syngeneic host, reaching a size of about $10 \times 10$ mm diameter in a mean time of $112 \pm 10$ days. Often the tumour-bearing animals were still alive 1 year after inoculation. During the subsequent transplants all the tumours grew progressively faster: 17 of them changed into sarcomata by the 5th transplant, whereas the other 19 retained their initial adenomatous morphology at the 10th transplant generation (Fig. 1). In most instances the morphological modification was abrupt, although on several occasions admixtures of glandular structures and a solid growth resembling sarcoma were observed (Fig. 2, 3 and 4). Five of the 19 tumours which were found to retain the adenomatous morphology at the 10th passage, were studied also at subsequent transplants and no changes in morphology were noted (Fig. 5).

To exclude the possibility that the sarcomatous change was due to a recruitment of mesenchymal host cells from the fibrous capsule surrounding tumours, a primary adenoma from the most immunodepressed group of treatment (D)

FIG. 1.—Change of morphology of 36 lung adenomata during serial s.c. transplants.

FIG. 2.—Typical adenomatous structure of a lung adenoma at the 1st s.c. transplant. × 150.

FIG. 3.—A transition form between adenomatous and sarcomatous morphology at the 2nd s.c. transplant. × 375.

FIG. 4.—Sarcomatous structure of a lung adenoma at the 8th s.c. transplant. × 375.

FIG. 5.—Adenomatous structure of a lung adenoma at the 12th s.c. transplant. × 150.

was serially transplanted s.c. in hybrid (C3Hf × BALB/c)$F_1$ mice, where at the 3rd transplant generation it acquired a sarcomatous structure. The tumour was retransferred into the 2 parental strains, C3Hf and BALB/c, at the 1st, 3rd, and 5th transplant, and was found to grow in BALB/c mice only (Table I).

To rule out a possible role of the subcutaneous location of the tumour transplants, another primary adenoma of Group D was serially injected as well into the tail vein so that the adenomatous cells could reach the lung and grow in their original milieu. The tumour acquired a sarcomatous morphology with both techniques, at the 3rd passage when transplanted s.c. and at the 4th passage

TABLE I.—*Growth of a BALB/c Lung Adenoma in (C3Hf × BALB/c)$F_1$ Hybrids and Subsequent Transfer in the Parental Mice*

| | Tumour growth in (C3Hf × BALB/c)$F_1$ | | | Transfer in | | | |
|---|---|---|---|---|---|---|---|
| | | | | C3Hf | BALB/c | | |
| Tranaplant generation | Tumour take* | No. of days to a 10 × 10 mm tumour† | Morphology | Tumour take* | Tumour take* | No. of days to a 10 × 10 mm tumour† | Morphology |
| 1 | 4/4 | 40 | adenoma | 0/4 | 4/4 | 42 | adenoma |
| 2 | 2/3 | 47 | adenoma | NT‡ | NT‡ | | |
| 3 | 4/4 | 25 | sarcoma | 0/4 | 3/3 | 25 | sarcoma |
| 4 | 4/4 | 20 | sarcoma | NT‡ | NT‡ | | |
| 5 | 4/4 | 19 | sarcoma | 0/4 | 4/4 | 20 | sarcoma |

* No. of mice with tumour/No. of transplanted mice.

† Mean value for the growing tumours.

‡ Not tested.

when growing in the lung. The tumour cells injected i.v. originated numerous small nodules in all lobes.

*Growth and metastasis characteristics*

The subcutaneous growth of 5 lung adenomata which did not change their morphology after serial passages and of 8 which transformed into sarcomata, was evaluated from the 1st through the 8th transplant generation. As shown in Fig. 6, tumour growth, expressed as the time taken to reach a 10 × 10 mm diameter, was accelerated in the successive transplants for both tumour types, though the sarcomatous tumours grew more rapidly then the adenomatous ones.

Six tumours, 3 adenomatous and 3 sarcomatous, were also studied at various transplant generations for their ability to give rise to lung metastases, within 2, 4, 6 or 8 weeks after s.c. implantation. The mice came under observation either on spontaneous death or on sacrifice when moribund or at the time scheduled for killing. Since no differences were noted at the different transplants, the data for each tumour were pooled (Table II). Two sarcomatous tumours killed the host before the 6th or the 8th week with only 1 case of lung metastases, death being ascribed to the huge s.c. growth. One sarcomatous and 2 adenomatous tumours gave pulmonary metastases, the former earlier and at a higher rate than the latter. The animals implanted with the 3rd adenomatous tumour did not develop metastases.

Fig. 6.—Relationship between morphology and growth rate of lung tumours during s.c. transplantation: □——□ adenomatous, ■——■ sarcomatous tumours.

*Correlation between antigenicity and subsequent morphology*

Nine lung adenomata were tested at the first transplant, when all were adenomatous, for membrane antigenicity by an *in vitro* microtest for cell-mediated immunity. Five tumours were destroyed by lymphocytes immune against a syngeneic lung adenoma previously demonstrated immunogenic, and all of them acquired the sarcomatous morphology during the subsequent transplants. Four tumours were negative for membrane antigenicity and all of them maintained the adenomatous appearance permanently (Table III). Eleven tumours were tested by immunodiffusion for the presence of

Table II.—*Capability of Adenomatous and Sarcomatous s.c. Tumours to Give Rise to Lung Metastases*

| No. of mice | Tumour type | Treatment group | Transplant generation | Animals with lung metastases/Total: 2 weeks | 4 weeks | 6 weeks | 8 weeks |
|---|---|---|---|---|---|---|---|
| 42 | sarcoma | C | 8–15 | 0/5 | 2/18 | 17/19 | no survivors |
| 32 | sarcoma | C | 10–20 | 0/12 | 1/20 | no survivors | no survivors |
| 36 | sarcoma | B | 9–17 | 0/4 | 0/26 | 0/6 | no survivors |
| 38 | adenoma | B | 6–12 | NT* | 0/14 | 0/10 | 2/14 |
| 37 | adenoma | A | 12–17 | 0/3 | 0/16 | 0/9 | 0/9 |
| 29 | adenoma | C | 14–17 | NT* | 0/10 | 0/9 | 3/10 |

* Not tested.

TABLE III.—*Relationship Between Morphology and Antigenicity of* 12 *Lung Adenomata**

| Tumours | Treatment group | Final morphology | No. of cells remaining after incubation with lymphocytes ± SE: Immune | Normal | % reduction | gs antigen |
|---|---|---|---|---|---|---|
| Ad 1 | D | sarcoma | 38±9 | 74±13 | 49† | + |
| Ad 14 | C | sarcoma | 134±9 | 213±12 | 38† | + |
| Ad 18 | C | sarcoma | NT‡ | NT‡ | | + |
| Ad 24 | A | sarcoma | 122±10 | 187±13 | 35† | NT‡ |
| Ad 26 | B | sarcoma | NT‡ | NT‡ | | – |
| Ad 44 | B | sarcoma | 55±6 | 114±19 | 52† | + |
| Ad 62 | D | sarcoma | 114±8 | 224±11 | 49† | + |
| Ad 29 | B | adenoma | 93±13 | 87±10 | +7 | – |
| Ad 35 | A | adenoma | 124±16 | 137±12 | 10 | – |
| Ad 36 | A | adenoma | 32±5 | 36±6 | 12 | – |
| Ad 50 | B | adenoma | NT‡ | NT‡ | | – |
| Ad 54 | C | adenoma | 116±9 | 122±9 | 5 | – |

* The cytotoxicity test was done on tumours at the first transplant, the gs test on transplanted tumours when they had reached the definitive morphology.
† $P < 0{\cdot}05$.
‡ Not tested.

the gs antigen when they had acquired the final morphology; 5 out of 6 sarcomatous tumours tested were positive at dilutions ranging between 1:32 and 1:128 whereas all 5 adenomatous ones were negative, even when tested undiluted (Table III).

*Correlation between immunological status of the primary host and subsequent morphology*

The 36 lung adenomata used in the present study were originally induced in 4 groups of mice with 4 different carcinogenic schedules of treatment which had a different immunodepressive effect, and their antigenicity as primary tumours correlated directly with the immune status of the host, *i.e.* the more immunodepressed hosts originated the more antigenic tumours (Colnaghi *et al.*, 1971; Ménard *et al.*, 1973). As shown in Fig. 7, we now report a correlation between the immunological status of the original host and the proneness of the tumours to change morphology, since almost all the tumours that arose in the most immunodepressed group became sarcomatous, whereas about 75% of the tumours arising in the less immunodepressed group remained adenomatous.

FIG. 7.—Relationship between immunodepression of the primary tumour host, evaluated by the Jerne technique, and the proneness of 36 lung adenomata to become sarcomatous after 7 transplant generations. 8–12 tumours per group of treatment: A and B, low and high dose of urethane, C and D, urethane plus cortisone.

### DISCUSSION

The change of morphology of lung adenomata during successive transplant generations is an already reported but unexplained phenomenon, occurring in about 50% of the transplanted tumours (Stewart, 1959). We studied various parameters of this phenomenon and the results suggest that the progression from a glandular to a sarcomatous pattern is not a random but a predetermined event.

All the lung tumours studied were adenomatous at the first transplant when they grew slowly, in general without killing the host. We found that, upon serial transplantation, some tumours changed their histologic morphology within a few transplants, becoming sarcomatous, whilst others retained their original adenomatous structure for as long as tested. The change of morphology does not seem to be influenced by the s.c. growth of the transplanted tumours, since the adenomata which eventually became sarcomatous after s.c. transplants, transformed also when allowed to grow in the lung. We can exclude a recruitment of mesenchymal host cells since a BALB/c lung adenoma transplanted in $F_1$ hybrids and then retransferred in the 2 parental strains, still grew well in the BALB/c host.

The difference in morphology was only partially correlated with the capacity of the tumours for growth or metastasis assumed as indicators of malignancy. The 2 morphologically different types of lung tumours in fact both increased their growth capacity in the successive transplants, although the sarcomatous tumours grew more rapidly than the adenomatous ones. Both types had the capacity to metastasize but the sarcomatous tumours metastasized more rapidly.

The immunological behaviour of the 2 types of tumour differed, since only tumours that changed their morphology after the first few transplants were found to possess tumour-associated membrane antigens at the first transplant. This antigenicity was conditioned by the immune responsiveness of the primary tumour host, since the majority of the antigenic adenomata were observed in the most immunodepressed groups, and seems to be related to a C-type oncornavirus, since a mouse anti-G serum was completely absorbed by antigenic adenomata (unpublished results). Moreover, only sarcomatous tumours were gs-positive, at least as revealed by the sensitivity of the assay we used.

It therefore appears that adenomata destined to become sarcomatous are somehow related to a viral activity. A- and C-type particles have been described in primary lung adenomata and it has been reported that A-particles disappeared upon transplantation whereas C-particles increased (Bucciarelli and Ribacchi, 1972). We suggest that a predisposition to sarcoma progression is related to a complex immunological control at the time of adenoma induction that conditions the activity of an oncorna virus which, although perhaps without an etiological role in adenomagenesis, is nonetheless responsible for the superimposed sarcomatous change of tumours occurring in immunodepressed animals. The change does not take place when the immune system of the primary tumour host can control the virus, either by modulating its antigenic expression, as already reported for cellular and viral antigens (Boyse *et al.*, 1967; Ioachim *et al.*, 1972), or by eliminating the antigenic cells.

This work was in part supported by a research grant from the Consiglio Nazionale delle Ricerche, Rome.

### REFERENCES

Boyse, E. A., Stockert, E. & Old, L. J. (1967) Modification of the Antigenic Structure of the Cell Membrane by Thymus-Leukemia (TL) Antibody. *Proc. natn. Acad. Sci.*, **58**, 954.

Bucciarelli, E. (1971) Isogeneic Transplants of Lung Tumors Induced by Hydrazine Sulfate in BALB/c/cb/Se Mice in Adult and Newborn Hosts: An Electron Microscopic Study. *Lav Ist. Anat. Istol. patol. Perugia*, **31**, 19.

Bucciarelli, E. & Ribacchi, R. (1972) C-type Particles in Primary and Transplanted Lung Tumors Induced in BALB/c Mice by Hydrazine Sulfate: Electron Microscopic and Immunodiffusion Studies. *J. natn. Cancer Inst.*, **49**, 673.

Colnaghi, M. I., Ménard, S. & Della Porta, G. (1971) Demonstration of Cellular Immunity Against Urethan-induced Lung Adenomas of Mice. *J. natn. Cancer Inst.*, **47**, 1325.

Ioachim, H. L., Dorsett, B., Sabbath, M. & Keller, S. (1972) Loss and Recovery of Phenotypic Expression of Gross Leukemia Virus. *Nature, New Biol.*, **237**, 215.

Kimura, I., Miyake, T., Ishimoto, A. & Ito, Y. (1972) Intracisternal A-type and C-type Particles Observed in Pulmonary Tumors in Mice. *Gann*, **63**, 563.

MÉNARD, S., COLNAGHI, M. I. & CORNALBA, G. (1973) Immunogenicity and Immunosensitivity of Urethane-induced Murine Lung Adenomata, in Relation to the Immunological Impairment of the Primary Tumour Host. *Br. J. Cancer*, **27**, 345.

STEWART, H. L. (1959) In *Physiopathology of Cancer*. Ed F. Homburger and W. Fishman. pp. 18–37. Karger.

STEWART, H. L., GRADY, H. G. & ANDERVONT, H. B. (1947) Development of Sarcoma at Site of Serial Transplantation of Pulmonary Tumors. *J. natn. Cancer Inst.*, **7**, 207.